# A novel giant non-cholinergic striatal interneuron restricted to the ventrolateral striatum coexpresses Kv3.3 potassium channel, parvalbumin, and the vesicular GABA transporter

**DOI:** 10.1038/s41380-020-00948-4

**Published:** 2020-11-14

**Authors:** Lydia Lebenheim, Sam A. Booker, Christian Derst, Torsten Weiss, Franziska Wagner, Clemens Gruber, Imre Vida, Daniel S. Zahm, Rüdiger W. Veh

**Affiliations:** 1 Institut für Integrative Neuroanatomie, Charité-Universitätsmedizin Berlin, Philippstraße 12, D-10115, Berlin, Germany; 2 Department of Pharmacology and Physiology, Saint Louis University School of Medicine, 1402 S. Grand Blvd, Saint Louis, MO 63104 USA; 3 Institut für Zell- und Neurobiologie, Charité -Universitätsmedizin Berlin, Philippstraße 12, D-10115, Berlin, Germany; 4 Present Address: Centre for Discovery Brain Sciences, University of Edinburgh, Edinburgh, EH8 9XD UK; 5 Present Address: Hans Berger Klinik für Neurologie, Universitätsklinikum Jena, An der Klinik 1, D-07747, Jena, Germany

**Keywords:** Neuroscience, Physiology

## Abstract

The striatum is the main input structure of the basal ganglia. Distinct striatal subfields are involved in voluntary movement generation and cognitive and emotional tasks, but little is known about the morphological and molecular differences of striatal subregions. The ventrolateral subfield of the striatum (VLS) is the orofacial projection field of the sensorimotor cortex and is involved in the development of orofacial dyskinesias, involuntary chewing-like movements that often accompany long-term neuroleptic treatment. The biological basis for this particular vulnerability of the VLS is not known. Potassium channels are known to be strategically localized within the striatum. In search of possible molecular correlates of the specific vulnerability of the VLS, we analyzed the expression of voltage-gated potassium channels in rodent and primate brains using qPCR, in situ hybridization, and immunocytochemical single and double staining. Here we describe a novel, giant, non-cholinergic interneuron within the VLS. This neuron coexpresses the vesicular GABA transporter, the calcium-binding protein parvalbumin (PV), and the Kv3.3 potassium channel subunit. This novel neuron is much larger than PV neurons in other striatal regions, displays characteristic electrophysiological properties, and, most importantly, is restricted to the VLS. Consequently, the giant striatal Kv3.3-expressing PV neuron may link compromised Kv3 channel function and VLS-based orofacial dyskinesias.

## Introduction

The basal ganglia comprise a group of subcortical nuclei forming neuronal connectional loops beginning and ending in the cortex, via relays in the caudate putamen, pallidum, and thalamus [1]. Basal ganglia are well known for their contribution to voluntary movement generation [2, 3]. Neurological disorders, including Parkinson’s and Huntingtons’s disease, arise from pathologies in these circuits [4]. Additionally, the basal ganglia contribute to behavioral patterns [5] and emotional states [3]. Consequently, the dysfunction of basal ganglia circuits also has been reported in psychiatric disorders [6–9].

The main input receiving structure of the basal ganglia is the striatum consisting of the caudate nucleus and the putamen, which in higher mammals, but not rodents, are separated by the internal capsule. The striatum is divided into functionally distinct subfields. The lateral striatum is the input region for sensorimotor loops and reflects the topography of the cortical homunculus. The ventrolateral striatum (VLS) is the orofacial target field of that homunculus with functional relevance for food intake related, reward-associated behavior, and for facial expressions corresponding to emotions [10–13]. Inputs to the VLS include fibers from temporal lobe, sensorimotor cortex, amygdala, and perirhinal and insular areas relevant to food intake and taste [14–16]. Orofacial dyskinetic and dystonic movements common to several CNS diseases and drug-induced syndromes, including tardive dyskinesia [17, 18], most likely arise from dysfunction of circuits involving the VLS. A rodent model presenting vacuous chewing movements due to systemic administration of neuroleptic drugs [19] is often used to study tardive dyskinesia, a side effect of neuroleptic treatment. Additionally, pilocarpine, amphetamine, physostigmine, and acetylcholine directly injected into the VLS, but not other stiatal regions, induce vacuous chewing movements [17, 18, 20, 21]. General abnormalities in all major neurotransmitter systems of the striatum including GABA [22–24], acetylcholine [25, 26], and dopamine [27] fail to explain the prominence of orofacial side effects following neuroleptic treatment.

To better understand the special vulnerability of the VLS to neuroleptic drugs, the present study aimed to recognize molecular features that distinguish the VLS from other striatal areas. In this regard, potassium channels display highly characteristic distributions in the brain and, especially, in the striatum [28–30], suggesting that they may subserve highly specific biological functions. In this regard, the Kv2.2 potassium channel protein and a channel-related protein (KChIP3) recently have been used as markers for characteristic cells in the brain [31, 32].

Consequently, the present investigation is concerned with the distribution of Kv channel proteins in the striatum, with a view to establishing links between these ion channels and disturbed general motor processes within VLS, the striatal area specifically involved in orofacial movements. The distribution of voltage-gated (Kv) potassium channels within the striatum is less well known than is that of, e.g., inwardly rectifying or other potassium channels. Disturbed Kv channel activity is associated with motor dysfunction, neurodegeneration, and developmental deficits. Thus, mice with double knockout of Kv3.1 and Kv3.3 display a motor phenotype [33–35]. Mutations of the KCNC3 gene (Kv3.3 channel protein) are involved in the adult-onset spinocerebellar ataxia SCN13 [36, 37], and dysfunction in Kv1 subunits may cause episodic ataxia [38, 39]. Autoantibodies against Kv channels are involved in Isaac’s syndrome [40] and mutated Kv3 family channels may cause neuronal death [37]. The expression of Kir2.1, Kir2.3, and Kv2.1 channels is decreased in striatal medium-sized spiny neurons [41] and inwardly and outwardly rectifying K+ currents are reduced in mouse models of Huntington’s Disease [36].

## Materials and methods

A more detailed version of the “Materials and methods” section is available as Supplementary information.

### Chemicals

Chemicals were obtained from Sigma, Taufkirchen, Germany, if not indicated otherwise.

### Animals

All animal experiments were approved by the Regional Berlin Animal Ethics Committee and conducted in strict accordance with the European Communities Council directive regarding care and use of animals for experimental procedures. Adult (250–300 g) male (5) and female (5) Wistar rats, male Sprague Dawley (3) and Long Evans (2) rats and 5 male adult C57BL/6 mice were obtained from an institutional breeder (Department for Experimental Medicine (FEM), Charité University Medicine Berlin). Three rats expressing the Venus fluorescent protein under the promotor of the vesicular GABA transporter (vGAT1) were bred on a Wistar background [42]. Animals were group-housed under controlled temperature (22 °C) and illumination (12 h cycle) with water and food ad libitum. A forebrain block of an adult male mouse (K-01/489) and striatal sections of an adult male rhesus monkey (K-06/108) were available from earlier investigations. A total number of 22 animals was included in the present experiments. None of the selected animals subsequently was excluded from experiments. No randomization of subjects was done.

### Whole-cell patch-clamp recordings in acute slice preparations

Diencephalic slices were prepared as previously described [43]. For whole-cell patch-clamp recordings, slices were transferred to a submerged recording chamber perfused with carbogenated ACSF (in mM: 125 NaCl, 2.5 KCl, 25 NaHCO_3_, 1.25 NaH_2_PO_4_, 25 glucose, 1 MgCl_2_, 2 CaCl_2_, 1 Na-pyruvate, 1 Na-ascorbate) maintained at 32 ± 1 °C with an inline heater (SuperTech, Switzerland) at a high flow rate of 10–12 ml/min. Slices were visualized with infrared differential contrast illumination (BX-50, Olympus, Hamburg, Germany) under a ×40 water-immersion objective (N.A. 0.8). Epifluorescent illumination (480 nm) of Venus fluorescent protein expressed under the vGAT promoter was used to pre-select giant VLS neurons, which, in turn, were filled with biocytin during patching. Further details have been described previously [43].

### Visualization, imaging, and reconstruction of the recorded neurons

Recorded neurons were identified post hoc as previously described [43]. Briefly, following successful outside-out patch formation, slices were fixed with 4% paraformaldehyde (PFA) in 0.1 M phosphate buffer (PB) overnight at 4 °C, washed in PB and phosphate-buffered saline (PBS; 25 mM PB, 150 mM NaCl, pH 7.4), and then blocked with 10% normal goat serum (NGS), 0.3–0.5% TritonX-100, and 0.05% NaN_3_ all diluted in PBS at room temperature for 1 h. Tissue-bound biocytin was visualized with the Elite-ABC-Complex as described below (see section “Immunocytochemistry”).

For multiple staining experiments, slices were incubated in primary antibodies (rabbit anti choline acetyltransferase (ChAT), 1:1000, Chemicon, USA; mouse anti parvalbumine (PV), 1:5000, Swant, Switzerland) followed by fluorescent secondary antibodies (Alexa Fluor 488 anti-rabbit IgG; Alexa Fluor 546 goat anti-mouse IgG, both 1:500; Invitrogen, Dunfermline, UK) and with fluorescently conjugated streptavidin (Alexa Fluor 647; 1:500, Invitrogen). Washed slices were mounted on glass slides with a polymerizing mounting medium (Fluoromount-G, Southern Biotech, AL, USA) and a 300 µm agar spacer and coverslipped.

Neurons were imaged with a confocal laser scanning microscope (FluoView 1000, Olympus, Hamburg, Germany) with a ×20 objective (N.A. 0.75; Olympus). Image stacks were collected at a resolution of 1024 × 024 or 2048 × 2048 pixels in the *xy*-plane and 0.5 or 1 µm steps between imaging planes along the *z*-axis. For selected neurons, high-resolution images were obtained with a ×60 silicone oil-immersion objective (N.A. 1.3) at 2048 × 2048 resolution and 0.05–0.1 µm steps between imaging planes in the *z*-axis. Pinhole was always set to 1 Airy unit [42]. Neurons were reconstructed offline from digitally stitched image stacks, which were segmented and reconstructed using semi-automatic analysis software [44]; Simple Neurite Tracer plug-in for the ImageJ/FIJI software package; http://fiji.org].

### Perfusion fixation

Rats were deeply anaesthetized by intraperitoneal injections of a cocktail consisting of 45% ketamine (100 mg/ml; Ketavet), 35% xylazine (20 mg/ml; Rompun) and 20% saline, at a dose of 0.16 ml⁄100 g of body weight, supplemented by 200 IU heparin i. p. to avoid clogging of brain vasculature during surgery. Subsequently, animals were fixed via transcardial perfusion with 4% PFA, 0.05% glutaraldehyde, and 0.2% picric acid in 0.1 M PB, pH 7.4 [45]. Brains were removed, cryoprotected in 0.4 M sucrose for about 4 h and in 0.8 M sucrose overnight, cut into blocks at preselected rostrocaudal levels, shock-frozen in hexane at −70 °C, and stored at −80 °C until used.

### Quantitative PCR

Four to six 20 µm thick cryostat sections from each of six perfusion fixed rat brains taken at the level of the anterior commissure were transferred to PBS. For probe generation, six samples of the dorsolateral and the VLS each were obtained by microscope assisted manual microdissection [46] of the sections. Samples were then transferred into small plastic tubes, frozen in liquid nitrogen, and kept at −80 °C. RNAs from perfusion fixed rat brain sections were isolated and quantitative PCR was performed using TaqMan assays (Applied Biosystems, Darmstdt, Germany) for raf Kv3.1 (Rn00563433_m1, spanning exon/intron boundary between exons two and three to exclude detection of genomic contamination) and rag Kv3.3 (Rn00588870_m1, spanning exon/intron boundary between exons one and two) as described earlier [47].

### In-situ-hybridization

For riboprobe preparation, cDNA regions containing the sequences of rat Kv3.1 (NM_012856.1, nucleotides 658–1185 and 2787–3118) and rat Kv3.3 (NM_053997.4, nucleotides 10–508 and 2021–2395) were PCR amplified using Advantage Taq Polymerase mixture (Clontech, Hamburg, Germany) and cloned into the pGEM-T vector (Promega, Karlsruhe, Germany). DIG-labeled sense and antisense riboprobes were prepared from SP6 and T7 promotor sites, respectively, using the DIG RNA Labeling Kit (Roche Diagnostics, Mannheim, Germany). In-situ-hybridization was performed as described earlier [47].

### Immunocytochemistry

Blocks of the brain containing the striatum were cut into 25 µm serial coronal sections on a cryostat, and pretreated as described earlier [48]. Primary antibodies (Table 1) were made in rabbit against Kv1.1 and Kv3.4 (authors laboratory), Kv1.3 (Prof. Knaus, Innsbruck), Kv3.1b, Kv3.2, and Kv3.3 (Millipore GmbH), Kv4.2 and Kv4.3 (Alomone Labs;), neuronal NO synthetase (Alexis biochemicals), ChAT (Chemion) and PV (Sigma-Aldrich GmbH). Mouse monoclonal antibodies were made against and neurofilament (NF) protein and SMI-32 (Sternberger monoclonals). Primary antibodies were followed by biotinylated secondary antibodies and visualized with the ABC complex as described earlier [48]. Long and short diameters of Kv3.3- or ChAT-stained neurons (30 cells each) were measured (on paper prints at 960-fold magnification without correction for tissue shrinkage or random orientation.

**Table 1:** Sources and dilutions of antibodies.

Antigen	Species	Monoclonal	Label	Source	Number	Dilution
Kv1.1 (KCNA1)	Rabbit	−	−	Author’s lab	R-94/55	1:10,000
Kv1.3 (KCNA3)	Rabbit	−	−	Author’s lab	R-02/59	1:50,000
Kv3.1b (KCNC1)	Rabbit	−	−	Chemicon	AB 5188	1:50,000
Kv3.2 (KCNC2)	Rabbit	−	−	Chemicon	AB 5190	1:10,000
Kv3.3 (KCNC3)	Rabbit	−	−	Chemicon	AB 5717	1:20,000
Kv3.4 (KCNC4)	Rabbit	−	−	Chemicon	AB 5192	1:20,000
Kv4.2 (KCND2)	Rabbit	−	−	Alomone labs	APC 023	1:100,000
Kv4.3 (KCND3)	Rabbit	−	−	Alomone labs	APC 017	1:10,000
NO synthase, neuronal	Rabbit	−	−	Sigma	N 2280	1:10,000
Choline acetyltransferase	Mouse	+	−	Chemicon	MAB5270	1:50,000
Parvalbumine	Rabbit	−	−	Sigma	P 3171	1:5,000,000
Neurofilament (SMI-32)	Rabbit	+	−	Sternberger monoclonals	SMI 32	1:50,000
Rabbit IgG	Goat	−	Biotin	Vector, Germany	BA-1000	1:2000
Mouse IgG	Horse	−	Biotin	Vector, Germany	BA-9500	1:2000

### Morphological stainings

Neurons alone or in combination with myelin sheaths were visualized with the Nissl and Klüver–Barrera staining procedures and acetylcholinesterase (AChE) enzyme activity was demonstrated as described earlier [49, 50].

### Photographic documentation

Sections were visualized with an upright Leica DMRB light microscope and images obtained with an Olympus SP-55UZ high-resolution digital camera. Primary photomicrographs were adjusted for background and brightness with Adobe Photoshop CS3 software (10.0) and the illustrations were produced with the aid of Adobe Illustrator CS3 software (13.0).

## Results

### The ventrolateral striatum is a morphologically distinct area in the mammalian striatum

**Fig. 1:**
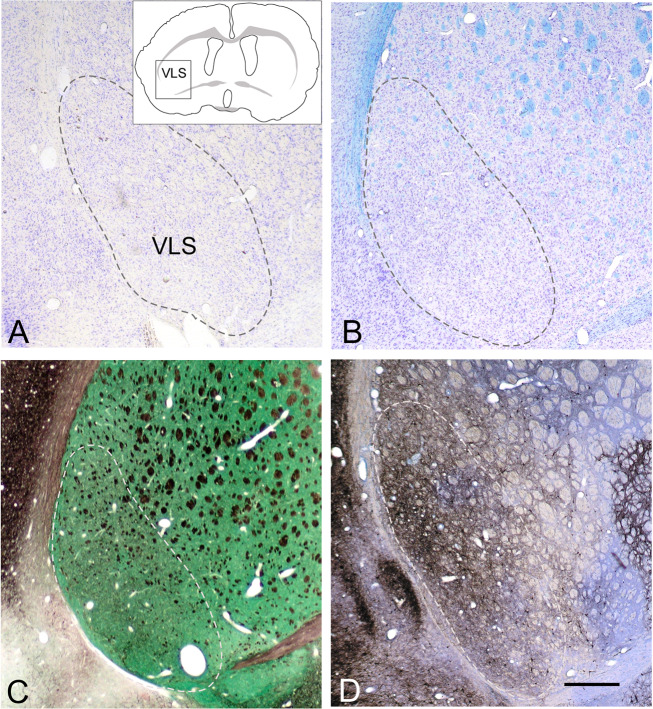
The ventrolateral striatum is a characteristic striatal subfield. The VLS (**A**, inset) is cell-dense with few fiber bundles as revealed by Nissl and Klüver–Barrera staining of overview micrographs (**A**, **B**; dashed lines) and visualization of acetylcholinesterase enzyme activity (**C**, green) and neurofilament immunostaining (**C**, black). VLS also contains fewer fiber bundles (**B**–**D**) and more parvalbumin immunoreactivity (**D**) than adjacent striatal subfields. Bar indicates 500 µm for all panels.

The VLS (boxed field in the inset of Fig. 1A) may already be recognized on morphological analysis following staining with cresyl violet or the Klüver–Barrera protocol as a cell-dense region with an inconspicuous medial border (Fig. 1A, B; dashed lines). It is distinguished from adjacent striatal areas by its higher cell density and low number of fiber bundles (Fig. 1C). The VLS presents as an oval field in the rat. In mice, it appears oval in the caudal striatum, but sickle shaped in the rostral striatum. The low density of fiber bundles leads to a characteristic appearance even in unstained tissue sections, which permits the identification of the VLS under the stereomicroscope to prepare DNA for quantitative PCR experiments.

In general, striatal interneurons are visualized after immunocytochemical staining for nitric oxide synthase (nNOS), PV, or combined staining for AChE and NF immunoreactivity. No characteristic details were seen with nNOS immunoreactivity and AChE enzyme activity alone (data not shown). The NF immunoreactivity highlights the low number of striatal fiber bundles and reveals a remarkable density of fine local collaterals in the VLS (Fig. 1C). Most strikingly, PV immunoreactivity is much stronger in the VLS as compared to adjacent striatal regions (Fig. 1D; Supplementary Fig. 1). This is not due to a greater density of PV neurons in the VLS, but rather to the fact that most of them are very big. Especially the local dendritic and axonal arborizations of PV neurons in the VLS are much denser as compared to PV neurons in other striatal areas, which are much smaller and have few local processes (Supplementary Fig. 1).

### Distribution of voltage-gated potassium channels in the VLS

**Fig. 2:**
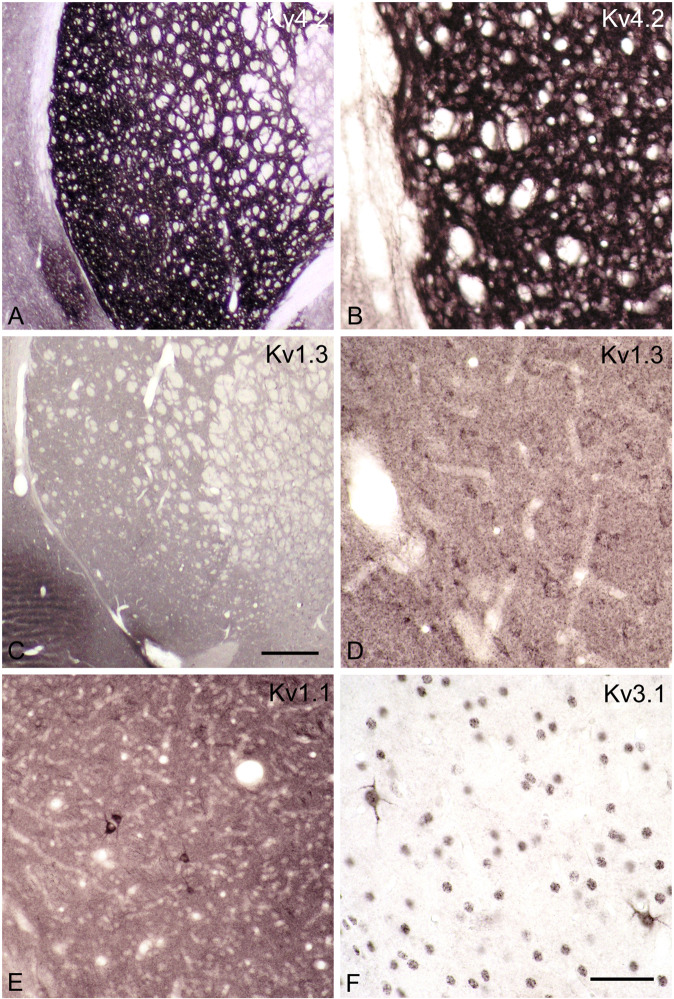
Members of the Kv potassium channel family are characteristically distributed in the striatum of the rat. Coronal sections through the rat forebrain are stained for Kv-immunorectivities using a peroxidase/DAB procedure. Kv4.2 immunoreactivity (**A**, **B**) is most prominent. The dense staining in the neuropil prohibits the visualization of individual neurons. Kv1.3 immunoreactivity is considerably weaker (**C**). Individual neurons can be detected at higher magnification (**D**). Staining for Kv1.1 (**E**) and Kv3.1 (**F**, residual nuclear reactivity is unspecific) is prominent in interneurons. Bar in (**C**) indicates 500 µm in (**A**–**C**), and bar in (**F**) indicates 100 µm in (**D**–**F**).

The distribution of individual potassium channel proteins, particularly members of the Kv1, Kv3, and Kv4 families, were analyzed as possibly novel anatomical or molecular attributes specific to the VLS. The expression of Kv4.2 immunoreactivity is very high in the striatum (Fig. 2A, B), especially in the neuropil, throughout the entire caudate putamen. Kv1.3 staining is considerably weaker (Fig. 2C, D) and mostly found in somata and dendrites of medium spiny neurons. Kv1.1 and Kv3.1 are expressed in very large neurons (Fig. 2E, F). With respect to size, distribution, and morphological appearance these cells strongly resemble and likely are identical to the very large Kv3.3-positive neurons described in the next paragraph. Thus, Kv1.1 and/or Kv3.1 may form heterooligomers with Kv3.3 in the VLS. Kv1.1/Kv3.3 heterooligomers have not been documented so far, but Kv3.1/3.3 herterooligomers are known [51]. Kv1.1 may be present in addition to such oligomers at different subcellular locations as has been documented for Kv1.1/1.2 oligomers and Kv3.4 in axon terminals of the cerebellar pinceau [52]. Kv3.2 and Kv3.4 are largely absent in the striatum (Supplementary Fig. 2A, B). Kv4.3 immunoreactivity is limited to presumed interneurons (Supplementary Fig. 2C, D), but whether cholinergic or GABAergic has not been investigated.

**Fig. 3:**
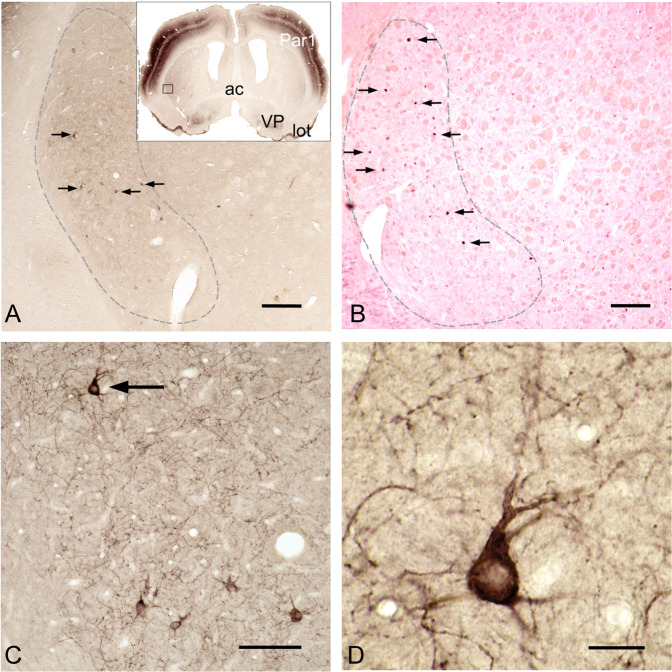
The VLS selectively has giant, Kv3.3-positive neurons. On survey micrographs of coronal sections through the rat forebrain (inset in **A**; the boxed area corresponds to **C**), Kv3.3 immunreactivity appears relatively weak in the VLS (**A**) as compared to the somatosensory cortex (Par1) or ventral pallidum (VP). Also note strong axon terminal staining in the terminal field of the lateral olfactory tract (lot). On closer inspection the Kv3.3-immunoreactive neuropil of the VLS (**A**, dashed line) is somewhat darker than surrounding areas due to intensely Kv3.3-immunoreactive neurons (**A**, black arrows) and their processes. In situ hybridization with an antisense Kv3.3 probe reveals an intense Kv3.3 mRNA signal in neurons in the same striatal area (**B**, dashed line). At higher magnifications, these are recognizable as very large Kv3.3-immunoreactive neuronal cell bodies with stout, early branching dendrites (**C**, **D**). Note the patchy distribution of Kv3.3-positive axons in the striatal matrix (**C**). Arrowed neurons in **A** correspond to those shown in **C**. The neuron arrowed in **C** is further enlarged in **D**. Bars indicate 250 µm in (**A**, **B**), 100 µm in (**C**), and 20 µm in (**D**).

Surprising, however, is the highly specific regional and cellular distribution of Kv3.3 immunoreactivity (Fig. 3A, inset; Supplementary Fig. 1) in the striatum. In the VLS, immunoreactivity against Kv3.3 appears modest at low magnification when compared to the somatosensory cortex (Fig. 3A, inset). Detailed inspection, however, discloses the presence of very large Kv3.3-positive cells in the VLS (Fig. 3C, D) that exhibit Kv3.3 expression at the protein (Fig. 3A) and mRNA level (Fig. 3B). Quantitative PCR data (Supplementary Fig. 3) substantiate preferential Kv3.3 expression in the VLS as compared to the dorsal striatum. In all but one rat, Kv3.3 mRNA was more abundant in the VLS than in the dorsal striatum and in 5 out of 12 animals (rats 1, 4, 6, 7, and 12), the VLS contained more than twice as much Kv3.3 mRNA as compared to dorsal striatum.

The Kv3.3/PV coexpressing neuron is a novel type of giant striatal cell restricted to the VLS

Cellular localization of Kv3.3 protein in the VLS is restricted to very large neurons (Fig. 3C, D), which at first glance resemble giant cholinergic interneurons (but see below). Cell bodies are oval to triangular with the long diameter of 15.8 ± 2.1 µm (maximum 24 µm) and the short diameter of 10.4 ± 1.3 µm (*n* = 30), very close to the dimensions of cholinergic interneurons (length: 17.7 ± 2.8 µm, width: 9.8 ± 1.8 µm; *n* = 30). They bear three to five stout dendrites (Fig. 3D) with an arborization field diameter of 500–600 µm. Note the patchy distribution (Fig. 3C) of Kv3.3-positive processes in the striatal matrix, which may indicate specific interactions with selected input areas or cell groups in the VLS.

**Fig. 4:**
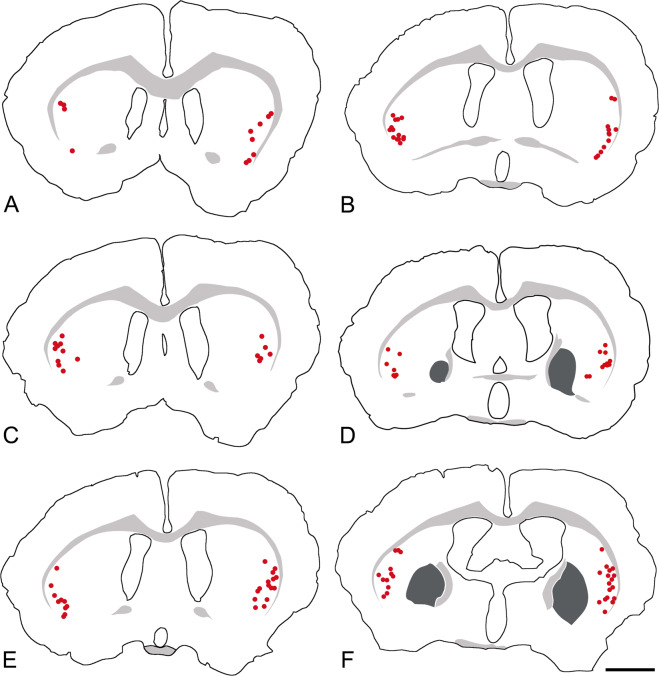
Giant, Kv3.3-positive neurons are restricted to the VLS along its entire rostrocaudal axis. The rostrocaudal distribution of the novel giant striatal neurons (NGSNs) in frontal sections spaced at 400 µm through the brain of a Wistar rat is shown schematically. Individual NGSNs are indicated by red dots. Each red dot represents one NGSN. Bar represents 2 mm for all sections.

Kv3.3-positive, novel giant striatal neurons (NGSNs) are present only in the VLS (Fig. 3A, B), the striatal area with the highest expression of PV (Fig. 1D, Supplementary Fig. 1). Double immunofluoescence reveals that PV and Kv3.3 are coexpressed in NGSNs (see below). The conspicuous restriction of Kv3.3-positive NGSNs to the VLS is most obvious when they are schematically displayed at several rostrocaudal levels along the complete rostrocaudal axis of rat brain (Fig. 4).

The presence of NGSNs in the VLS is not a spurious effect of breed, race, or species. Counts of Kv3.3-positive neurons in rats of different genders and strains (Wistar, Sprague Dawley, Long Evans) did not reveal any obvious variations in this pattern specific to a certain strain, to one of the hemispheres, or to the gender of the animals.

In both mouse and rat, as noted, Kv3.3 immunoreactivity appears weak in the VLS (Supplementary Fig. 4A, lower box) when compared to the somatosensory cortex (Supplementary Fig. 4A). At higher magnification it becomes apparent that the dorsomedial striatum (Supplementary Fig. 4A, upper box; enlarged in Supplementary Fig. 4B) is devoid of Kv 3.3-immunopositive cell bodies. In contrast, the mouse VLS (Supplementary Fig. 4A, lower box; enlarged in C) contains Kv3.3-immunopositive cells (Supplementary Fig. 4C, arrows), which at higher magnification (Supplementary Fig. 4D) much resemble those observed in the rat VLS (compare to Fig. 3D).

Our Kv3.3 antibody did not detect Kv3.3 channels unequivocally in monkey brain (Supplementary Fig. 4E), which, however, did exhibit very large, PV-positive neurons restricted to the VLS (Supplementary Fig. 4F), indicating that the NGSNs also are present in primates. Actually, “giant, PV neurons” have been described before in the monkey striatum, but without specifying any characteristic striatal localization [53].

### NGSNs are distinct from giant cholinergic interneurons

The NGSNs in the VLS morphologically resemble giant cholinergic striatal interneurons. However, they are restricted to the VLS, while giant cholinergic interneurons, also known as tonically active neurons (TANs), are found throughout the entire striatum. This fact already indicates that the Kv3.3-immunoreactive neurons, which also express PV and GAD immunoreactivity, are distinct from the cholinergic interneurons.

**Fig. 5:**
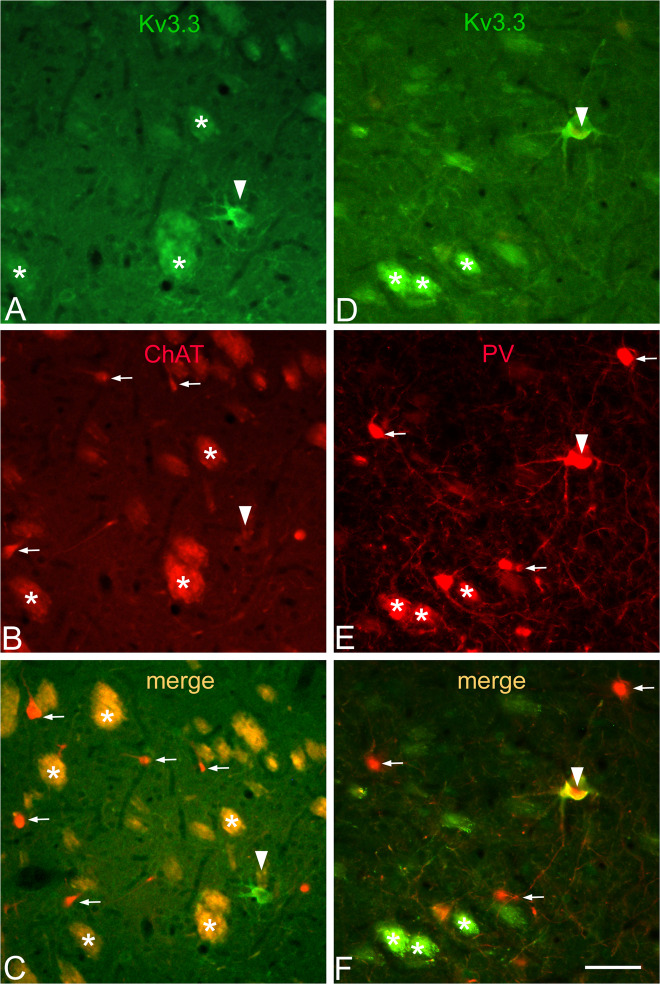
NGSNs are not cholinergic. A Kv3.3-immunopositive neuron in (**A**) is devoid of ChAT-immunoreactivity (**B**, arrowhead without target). After merging (**C**), red and green immunoreactive neurons remain distinct indicating no overlap of immunoreactivities. In contrast (**D**–**F**), a NGSN exhibits Kv3.3 (**D**, arrowhead) and parvalbumin (**E**, arrowhead) immunoreactivities (merge shown in **F**, arrowhead). In contrast, smaller parvalbumin-positive interneurons (**E**, **F**, arrows) do not exhibit the Kv3.3 immunoreactivity. Asterisks indicate unspecific staining in fiber bundles. Bar in (**D**) indicates 50 µm for all panels.

To support this, NGSNs in the VLS were subjected to co-immunolocalization of several transmitter markers. As expected, large Kv3.3-immunoreactive NGSNs (Fig. 5A, arrowhead) do not express ChAT, a cholinergic marker protein (Fig. 5B, arrowhead without target). Cholinergic interneurons have similar sizes and are immunoreactive for ChAT (Fig. 5B, C, arrows). After merge, Kv3.3-positive neurons remain green (Fig. 5C, arrowhead) and cholinergic cells remain red (Fig. 5C, arrows) indicating no overlap of immunoreactivitiy. In contrast, NGSNs, but not cholinergic neurons, display Kv3.3 (Fig. 5D, arrowhead) and PV (Fig. 5E, arrowhead) immunoreactivities (merge shown in Fig. 5F, arrowhead). Note also that small PV-positive cells (Fig. 5E, F, arrows) do not express the Kv3.3 channel.

Kv3.3/PV coexpressing NGSNs are distinct from giant cholinergic interneurons also by their electrophysiological properties

**Fig. 6:**
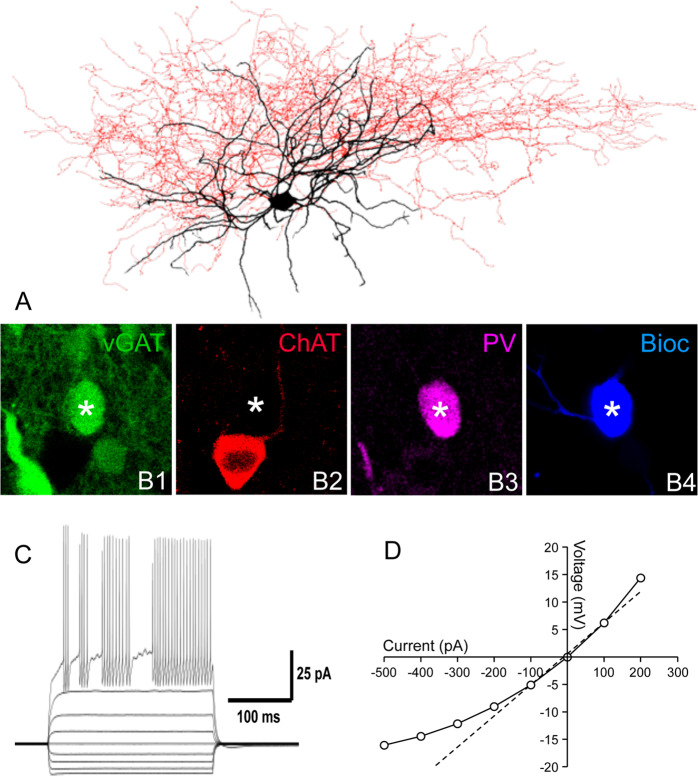
Electrophysiology discloses a fast-spiking phenotype for the NGSNs. A projected 3D-reconstruction of a NGSN from the VLS (**A**) shows the somatodendritic (black) and axonal (red) domains. The same cell is characterized by immunocytochemistry (B1–B4). Note the strong co-localization (asterisk) of biocytin labeling (Bioc) with vGAT-YFP (green pseudocolour) and also parvalbumin (PV, magenta pseudocolour), but not with ChAT (red pseudocolour). Note that the putative medium spiny neuron (B1, left lower corner) is vGAT positive but contains neither ChAT nor PV. A large ChAT positive cell (B2) is vGAT and PV negative. The NGSN in (**A**) responded to depolarizing current pulses (−500 to +1 nA, 500 ms) with fast spiking (**C**), as is typical for PV-containing interneurons. The current-voltage relationship of the same cell (**D**) discloses a low input resistance as well as a rectifying voltage response.

PV neurons in the striatum are known to be GABAergic and coexpress the vesicular GABA transporter vGAT. For a preliminary electrophysiological analysis, VLS NGSNs (*n* = 6) were identified in rat brain slices under video monitored infrared illumination by their typical localization in the VLS and their large sizes as mentioned above. Six cells were patch-clamped in the whole-cell mode, filled with biocytin (Fig. 6), and morphologically reconstructed (*n* = 3). As expected, the cells displayed very large somata with long, essentially smooth and sparsely branched dendrites. The distal parts of the dendrites showed numerous and prominent varicosities (Fig. 6A), which is atypical for cholinergic interneurons [54].

VLS NGSNs (*n* = 6) have a mean resting potential of −72.4 mV, similar to the value (−57.3, see Table 2) known for the large cholinergic interneurons [55]. In contrast, input resistance (86.8 MOhm versus 433.0 MOhm, respectively) and amplitude of after-hyperpolarization (−9.6 mV versus −21.8 mV, respectively) differ substantially between the NGSNs and cholinergic interneurons (Table 2). In addition, VLS NGSNs have a short half amplitude spike width (0.51 ms), which is considerably less than that of cholinergic interneurons (0.82 ms), but similar to the value found in fast-spiking interneurons (Table 2). Furthermore, the recorded VLS neurons displayed a spontaneous action potential discharge frequency of 220 ± 47 Hz, which is typical for fast-spiking interneurons. Altogether, the electrophysiological results support the view that the NGSNs in the VLS represent a novel type of fast-spiking striatal interneuron.

**Table 2:** Physiological properties of small, fast-spiking interneurons (SFS-INs) and the novel giant, fast-spiking interneurons (GFS-INs) from the ventrolateral striatum as found in the present report.

Intrinsic properties	SFS-IN (*n* = 4)	GFS-IN (*n* = 6)	GCH-INs
Resting membrane potential (mV)	−74.1 ± 1.7	−72.4 ± 3.6	−57.3 ± ??
Input resistance (MΩ)	64.6 ± 23.6	86.8 ± 30.5	433 ± ??
Membrane time constant (ms)	7.7 ± 1.6	10.2 ± 3.4	–
Action potential properties			
Amplitude (mV)	87.7 ± 7.4	90.7 ± 4.7	–
Threshold (mV)	−36.1 ± 4.9	−31.3 ± 6.7	–
Maximal rise/decay ratio	1.8 ± 0.2	2.13 ± 0.46	–
Half-height duration (ms)	0.38 ± 0.08	0.51 ± 0.12	0.82 ± 0.12
Instantaneous frequency (Hz)	283 ± 26	219 ± 47	–

Data of giant cholinergic interneurons (GCH-INs) are taken from the literature [55]. Data are given ± SEM.

## Discussion

### Morphological and functional aspects of the VLS

The VLS in rodents is distinct from other striatal areas. Based on morphological criteria, it is a cell-dense region with fewer fiber bundles than adjacent striatal areas (Fig. 1). Identification of the VLS is facilitated after combined staining for AChE enzyme activity and NF immunoreactivity (Fig. 1C) and particularly by its strong PV immunoreactivity (Fig. 1D; Supplementary Fig. 1) as has been reported [53–57].

Functionally distinct striatal subregions [21] like the VLS have long been known (see Introduction). Recent investigations support the potential importance of the VLS for involuntary orofacial movements. In monkeys with levodopa-induced dyskinesias (LID) the VLS was the only striatal region in which serotonin transporter ligand binding and mean diffusivity could be correlated with the LID-score [58]. Such data favor the concept of the VLS as a dyskinesia relevant subfield of the mammalian including primate striatum. In line with this idea, characteristic afferents differentiate the VLS from other striatal areas. Thus, it receives abundant projections from the temporal lobe, particularly from the amygdala and the perirhinal and insular cortices [15, 16, 59]. These cortical areas contain taste regions [60, 61] and the amygdala is involved with food intake and the processing of gustatory information [62]. Stimulation of the amygdala in monkeys results in orofacial movements. Patients with temporal lobe epilepsy suffer from oral automatisms and often experience gustatory or olfactory aurae [63].

The non-cholinergic vGAT/Kv3.3/PV coexpressing neuron (NGSN) is a novel cell type. So far, very large neurons in the striatum are known as giant, cholinergic, TANs [for review see [64]]. The NGSN described here is non-cholinergic and electrophysiologically distinct from TANs. Early Golgi studies demonstrated at least three distinct types of large neurons within the striatum [65]. The classical, large neurons with spindle-shaped, triangular or polygonal perikarya have long, essentially smooth and sparsely branched dendrites extending mainly from the poles of the cell (type 1). Other neurons with large round or oval somata have many, frequently branched, dendrites originating from all regions of the cell body giving the neuron a spidery appearance (type 2). Yet other neurons have very long (up to 700 µm) and essentially smooth dendrites (type 3). Importantly, the type 3 neurons were found only in the ventral part of the striatum [66]. The large neurons described by Bolam et al., however, were retrogradely labeled from the ipsilateral substantia nigra [65, 66], indicating that they are projection neurons. In addition, Bolam’s neurons were localized in a part of the ventral striatum, which does not correspond to the VLS. Thus, none of the three previously described large striatal neurons appears to be NGSNs reported in the present study.

### NGSNs represent a novel subclass of fast-spiking interneurons restricted to the VLS

In the striatum PV generally is coexpressed with GABA in medium-sized, aspiny interneurons [67, 68], which account for but a small percentage of striatal neurons. They are also known as fast-spiking interneurons [69] and have been shown to mediate feed-forward inhibition from the cortex [70]. The electrophysiological properties of PV interneurons depend on their potassium currents, which are due to members of the Kv3 family of potassium channels. These channels permit high-frequency firing, insofar as their voltage dependence and kinetic properties enable a fast reactivation of sodium channels. In addition, Kv3 currents cause brief action potentials and large after-hypopolarisation [71–73] thus limiting synaptic depression. PV striatal neurons have attracted much interest, as they apparently are involved in the generation of gamma oscillations, which were recently described in the ventral striatum [74].

Some heterogeneity of PV cells is known [70]. One subtype has a smaller soma and a relatively restricted and varicose dendritic arborization about 200–300 µm in diameter. The other displays a larger cell body and a more extended, non-varicose dendritic field of 500–600 µm in diameter. The latter type roughly resembles our Kv3.3-positive NGSNs, particularly with respect to the diameter of dendritic fields. In contrast, NGSNs possess clearly varicose dendrites (see Fig. 5E). Varicosities on dendrites of PV neurons are well known [55], supporting the idea that the non-cholinergic NGSNs described here belong to the group of PV cells. The larger subtype of PV cells, described in [70], had non-varicose dendrites, suggesting that they do not correspond NGSNs.

Irrespective of these issues, NGSNs most likely belong to the fast-spiking group of neurons. This hypothesis is in line with the morphology of these cells (see above) and is additionally supported by our electrophysiological data. The input resistance (86.8 MΩ) is close to that of fast-spiking interneurons (Table 2), and their spike width at half amplitude (0.51 ms) is well below 1 ms, which is the case in fast-spiking cells. Thus, based on our electrophysiological data NGSNs do not directly correspond to any of the known cell types in the striatum, but show strong similarity to the fast-spiking interneurons (Table 2).

The expression of Kv3.3-containing potassium channels by VLS NGSNs further supports this idea. In the neocortex, the coexpression of Kv3.1 and Kv3.3 subunits by some PV-positive interneurons is common [75], and the formation of Kv3.1/Kv3.3 heteromeric channels has been documented by co-immunoprecipitation [51]. In vivo, these cells are able to respond to sensory stimuli with high-frequency (>600 Hz) trains of action potentials [76]. NGSNs in the VLS display Kv3.1 immunoreactivity (Fig. 2E, F) in addition to Kv3.3-ir, supporting the idea of hetero-oligomeric Kv3.1/Kv3.3 channels in the novel VLS giant neurons.

The presence of NGSNs selectively in the VLS may provide a link between disturbed motor processes and an area specifically involved in orofacial movement.

Treatment of patients with schizophrenia with neuroleptics is the method of choice and often results in good outcomes and resocialization of the patients, which decreases hospitalization and keeps patients in the community. Long-term treatment, however, is jeopardized by severe side effects, including orofacial dyskinesias, which may bring treatment to an undesired end. Orofacial dyskinesia is a movement disorder that causes bizarre, rapidly repetitive movements of face and tongue. Even brief therapy with neuroleptic medications can cause this movement disorder, which is associated with abnormalities in all major neurotransmitter systems of the striatum, including GABA [22–24], acetylcholine [25, 26], and dopamine [27]. But this fact does not explain the orofacial preference of the motor side effects following neuroleptic treatment.

Defects of Kv3 channels have been linked to movement disorders and neurodegeneration. Although Kv3.3 mutant mice display no overt phenotype, Kv3.3 and Kv3.1 double-deficient mice show hyperactivity and increased locomotion and exploratory activity, spontaneous myocloni, and hypersensitivity to ethanol [37]. Mutations in KCNC3, the gene encoding the Kv3.3 potassium channel, are associated with CNS neurodegeneration and developmental deficits in humans. Especially, spinocerebellar ataxia SCN13 seems to be linked to Kv3.3 gene mutations [36]. Furthermore, Kv channels may be involved in the early stages of neurodegeneration in Alzheimer’s disease, consistent with the hypothesis that mutations of Kv3 family channels are sufficient to cause neuronal cell death [37]. NGSNs may provide a possible missing link, since NGSNs strongly express the Kv3.3 channel protein and are restricted to the VLS, an area with strong input from the orofacial sensorimotor cortex that repeatedly has been linked to orofacial dyskinesia [17, 18, 58].

One might ask the question, whether the discovery of NGSNs could help explicate the pathomechanisms of tardive dykinesia (TD) or orofacial dyskinesias (OFDs), or whether it may help to improve therapies. Yes, this may be the case. For example, the subunit composition of GABA-A receptors may differ in VLS PV neurons as compared to other striatal areas, which could permit more specific targeting of benzodiazepine drugs for the treatment of orofacial dyskinesia. Another important therapeutic is trihexyphenidyl (Artane), a selective blocker of the striatal M1-muscarinic receptor [M1Rs; [77], which mediates excitation of PV neurons [78] and consequent inhibition of striatal activity. NGSNs may express a higher amount of M1Ra. Insofar as NGSNs involvement in orofacial dyskinesia seems likely, Artane may be especially effective in the treatment of TD/OFD. Whether these pharmacological insights will bear fruit remains to be seen. What is clear from the present study is that the well known involvement of the VLS in TD/OFD is now firmly linked to the selective presence in VLS of NGSNs, which, because of the disproportionate role that fast-spiking, PV neurons play in striatal physiology, may well have an important role in the pathogenesis of TD/OFD, possibly involving the Kv3.3 channel.

## Supplementary information

Parvalbumin-positive neurons display distinct phenotypes in the ventrolateral and the dorsomedial striatum

Kv3 and Kv4 subunits are also expressed in the rat striatum

Kv3.3 mRNA is more abundant in the VLS as compared to the dorsal striatum

Also in the mouse NGSNs are restricted to the VLS
