# Supplementary figures and images for: A novel giant non-cholinergic striatal interneuron restricted to the ventrolateral striatum coexpresses Kv3.3 potassium channel, parvalbumin, and the vesicular GABA transporter
Source: Mol Psychiatry. 2020 Nov 14;27(4):2315–28. doi: 10.1038/s41380-020-00948-4 (PMC9126804; doi:10.1038/s41380-020-00948-4)

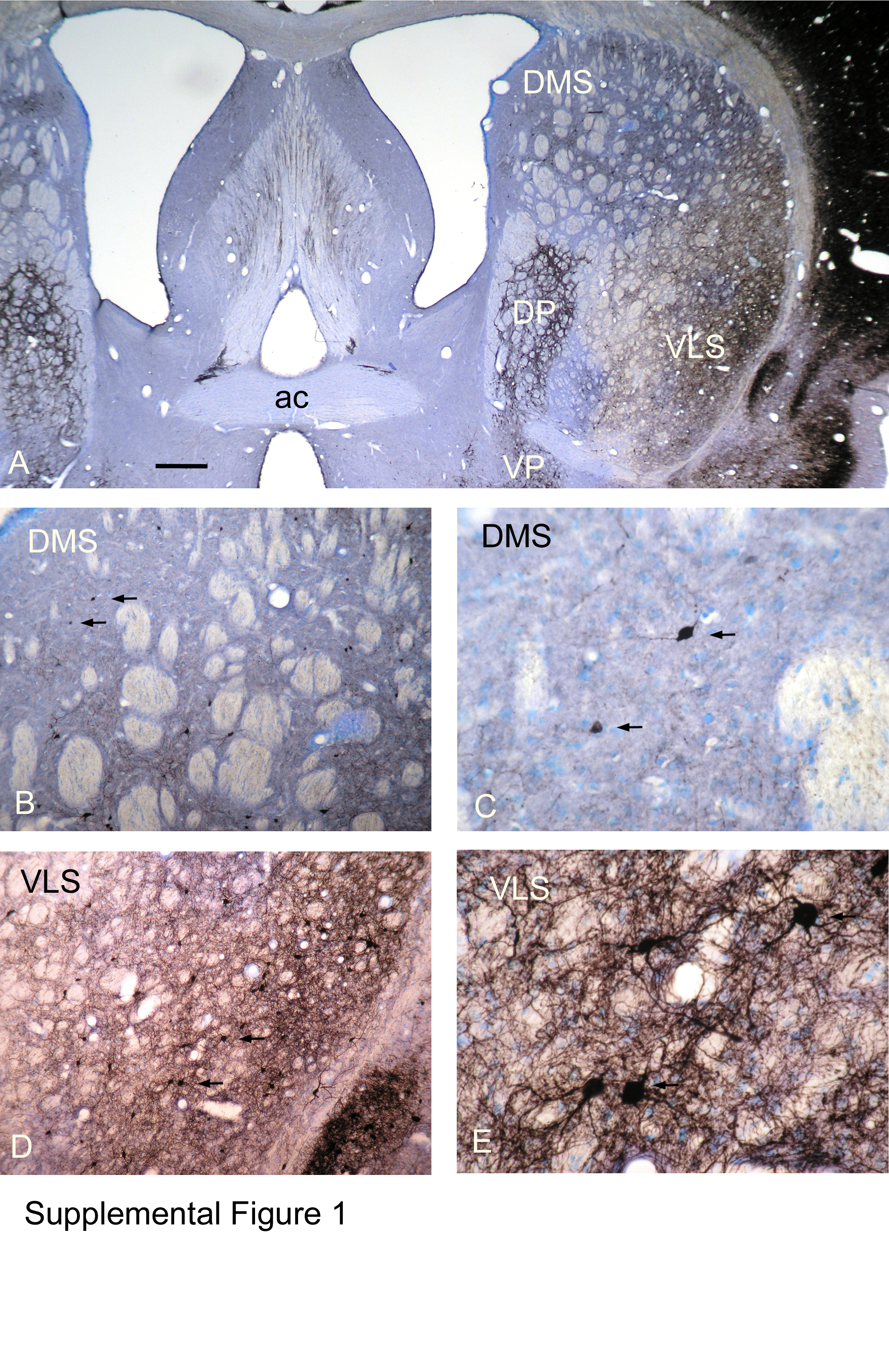

Supplement: Supplementary file 1 — Parvalbumin-positive neurons display distinct phenotypes in the ventrolateral and the dorsomedial striatum [file 41380_2020_948_MOESM1_ESM.jpg]

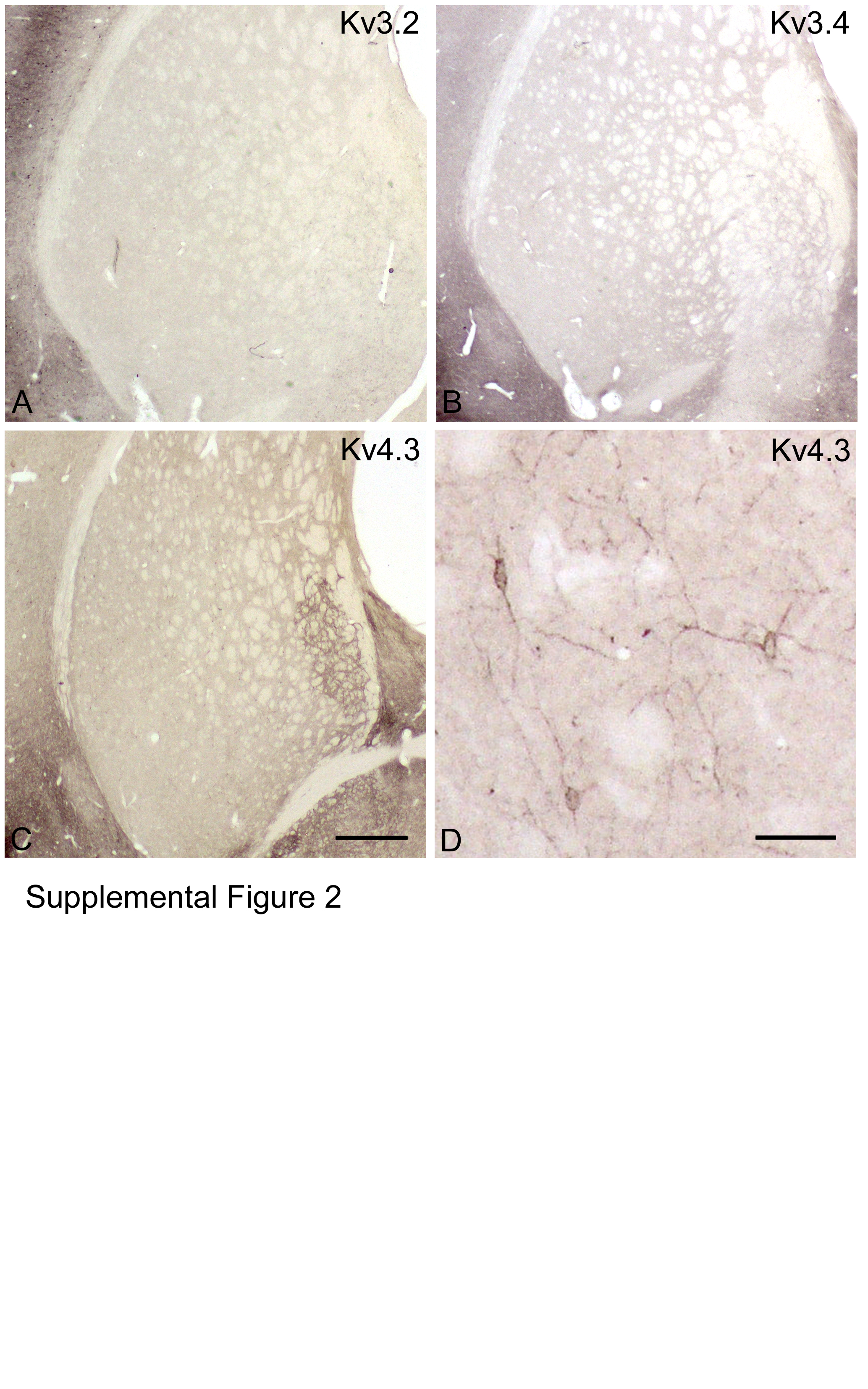

Supplement: Supplementary file 2 — Kv3 and Kv4 subunits are also expressed in the rat striatum [file 41380_2020_948_MOESM2_ESM.jpg]

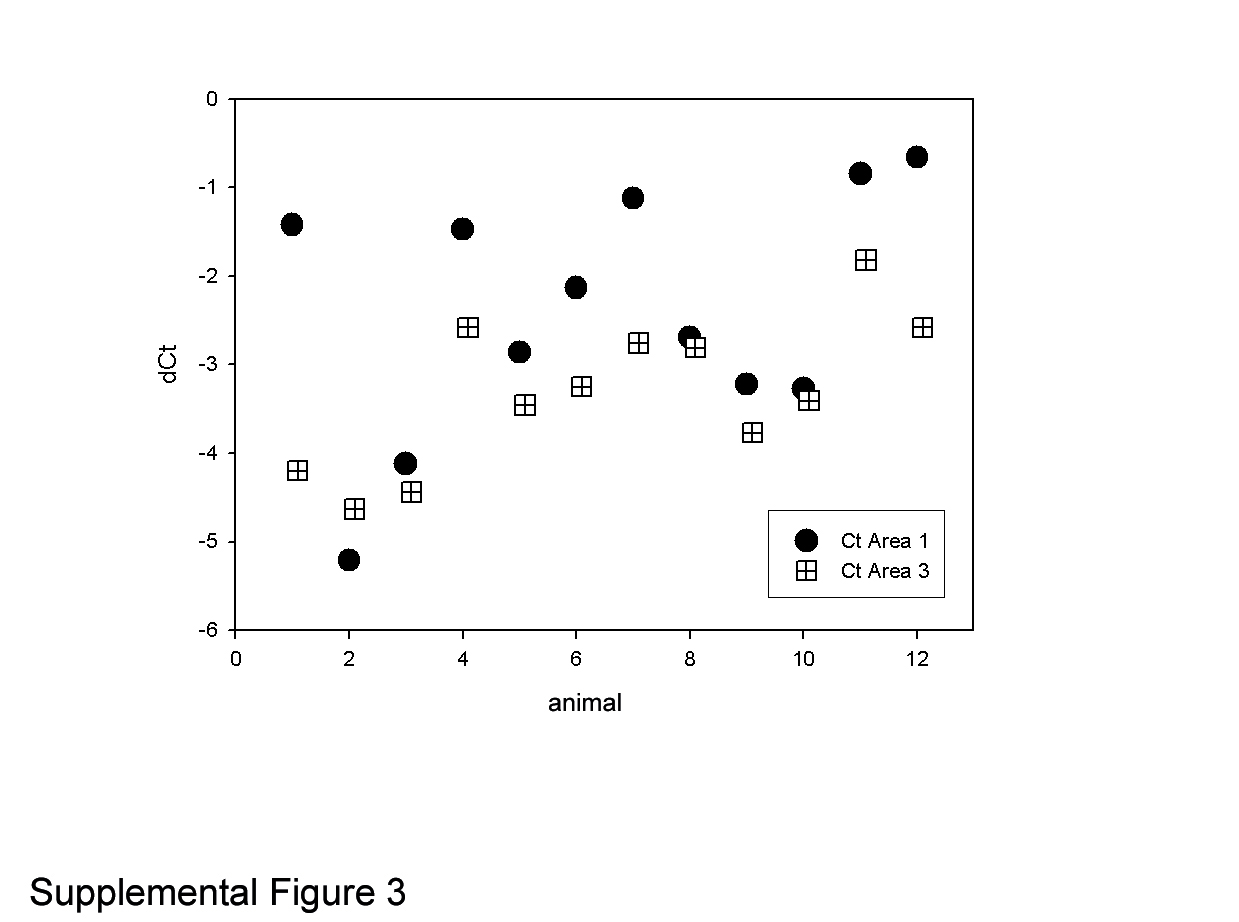

Supplement: Supplementary file 3 — Kv3.3 mRNA is more abundant in the VLS as compared to the dorsal striatum [file 41380_2020_948_MOESM3_ESM.jpg]

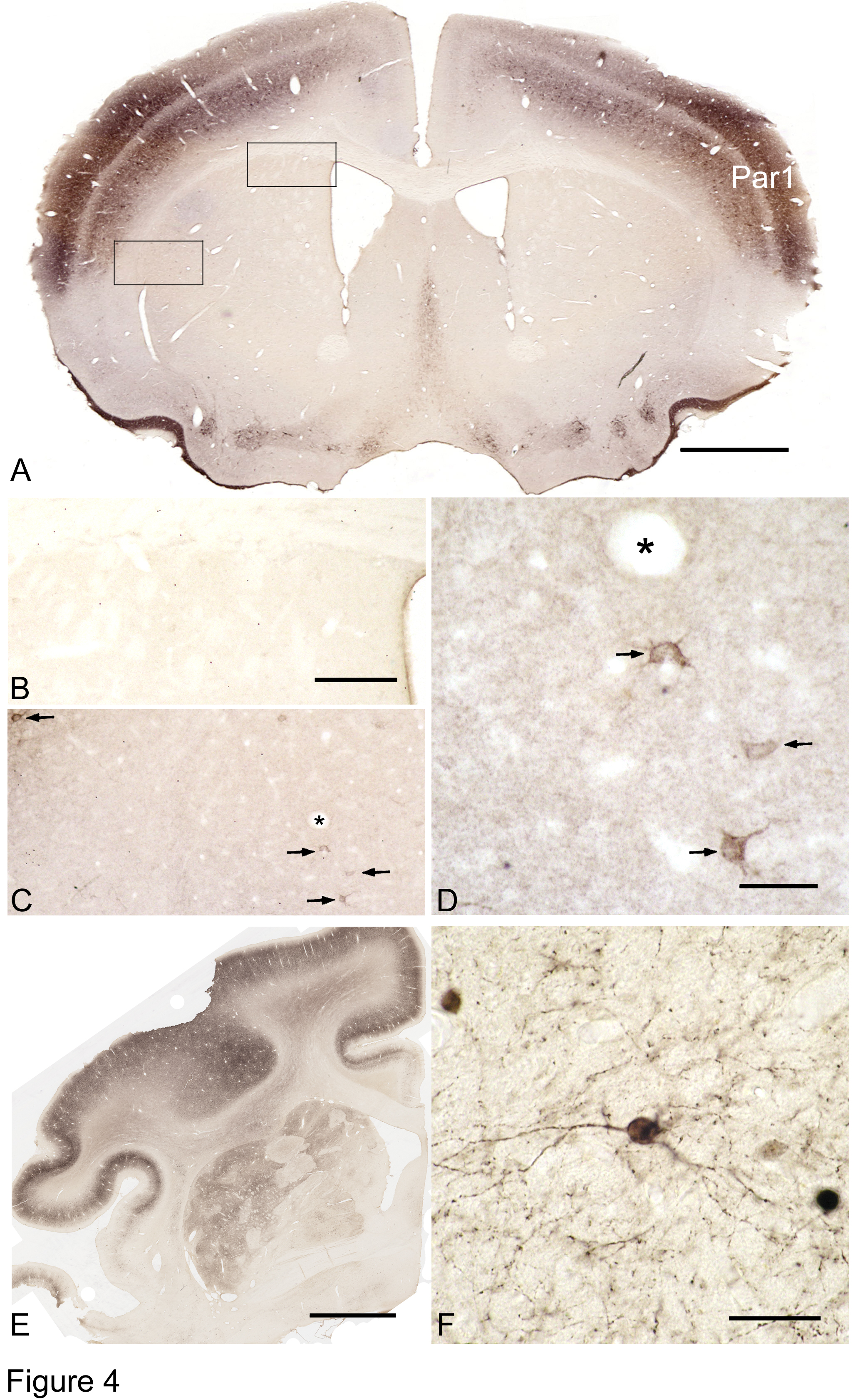

Supplement: Supplementary file 4 — Also in the mouse NGSNs are restricted to the VLS [file 41380_2020_948_MOESM4_ESM.jpg]
